# Selective improvement by rifaximin of changes in the immunophenotype in patients who improve minimal hepatic encephalopathy

**DOI:** 10.1186/s12967-019-2046-5

**Published:** 2019-08-28

**Authors:** Alba Mangas-Losada, Raquel García-García, Paola Leone, María Pilar Ballester, Andrea Cabrera-Pastor, Amparo Urios, Juan-José Gallego, Juan-José Martínez-Pretel, Carla Giménez-Garzó, Fernando Revert, Desamparados Escudero-García, Joan Tosca, María Pilar Ríos, Cristina Montón, Lucia Durbán, Luis Aparicio, Carmina Montoliu, Vicente Felipo

**Affiliations:** 1 Fundación Investigación Hospital Clínico, Instituto de Investigación Sanitaria, INCLIVA, Avda Menéndez Pelayo, 4acc, 46010 Valencia, Spain; 2 Laboratory of Neurobiology, Centro Investigación Príncipe Felipe, Valencia, Spain; 3 Unidad de Digestivo, Departamento Medicina, Hospital Clínico Valencia, Universidad Valencia, Valencia, Spain; 4 Servicio de Digestivo, Hospital Arnau de Vilanova, Valencia, Spain; 5 Departamento de Anatomía Y Embriología, Universidad Valencia, Valencia, Spain; 6 Departamento de Patología, Universidad Valencia, Valencia, Spain

**Keywords:** Minimal hepatic encephalopathy, Rifaximin, Immunophenotype, T lymphocytes activation

## Abstract

**Background:**

Minimal hepatic encephalopathy (MHE) in cirrhotic patients is associated with specific changes in parameters of the immune system reflecting a more pro-inflammatory environment than in patients without MHE. The aims of this work were to assess the effects of rifaximin treatment of cirrhotic patients with MHE on: (1) MHE; (2) intermediate (CD14^++^CD16^+^) pro-inflammatory monocytes; (3) expression of early activation marker CD69 in T lymphocytes; (4) autoreactive CD4^+^CD28^−^ T lymphocytes; (5) differentiation of CD4^+^ T lymphocytes to Th follicular and Th22; (6) serum IgG levels; and (7) levels of some pro-inflammatory cytokines.

**Methods:**

These parameters were measured by immunophenotyping and cytokine profile analysis in 30 controls without liver disease, 30 cirrhotic patients without MHE and 22 patients with MHE. Patients with MHE were treated with rifaximin and the same parameters were measured at 3 and 6 months of treatment. We assessed if changes in these parameters are different in patients who improve MHE (responders) and those who remain in MHE (non-responders).

**Results:**

Rifaximin improved MHE in 59% of patients with MHE. In these responder patients rifaximin normalized all alterations in the immune system measured while in non-responders it normalizes only IL-6, CCL20, and differentiation of T lymphocytes to Th22. Non-responder patients do not show increased expression of CD69 before treatment.

**Conclusions:**

Rifaximin normalizes changes in the immune system in patients who improve MHE but not in non-responders. Some alterations before treatment are different in responders and non-responders. Understanding these differences may identify predictors of the response of MHE to rifaximin.

## Background

A large proportion of patients with liver cirrhosis suffer covert or minimal hepatic encephalopathy (MHE), with attention deficits, psychomotor slowing and impaired visuo-motor and bimanual coordination which affect daily functioning and driving ability, reduces quality of life and life span, increases the number of falls, accidents and hospitalizations and predisposes to overt or clinical HE. MHE is therefore a serious health, social and economic problem [1–5].

The mechanisms leading to MHE involve the synergistic contribution of hyperammonemia and inflammation. In cirrhotic patients, inducing hyperammonemia impairs performance in psychometric tests during inflammation, but not after its resolution [6]. The serum levels of the pro-inflammatory cytokines IL-6 and IL-18 are higher in cirrhotic patients with MHE than in patients without MHE and correlate with cognitive impairment as assessed by the Psychometric Hepatic Encephalopathy Score (PHES) [7]. Also, the joint presence of hyperammonemia and inflammation above a certain threshold is enough to induce mild cognitive impairment even in the absence of liver cirrhosis. This occurs for example in some patients with non-alcoholic steatohepatitis (NASH) or in patients with keloids, without liver disease [8]. The contribution of peripheral inflammation to the cognitive and motor alterations in MHE has been clearly demonstrated in animal models. In rats with portacaval shunts, a model of MHE, reducing selectively peripheral inflammation by treating them with anti-TNFa, which does not cross the blood–brain barrier, prevents the induction of cognitive and motor alterations [9, 10].

We have recently performed a study to characterize the changes in peripheral inflammation associated to appearance of MHE, i.e. the alterations that are present in patients with MHE but not in patients without MHE [11]. Patients with MHE show a more pro-inflammatory environment in blood [11]. The main alterations associated specifically with MHE are: (1) increased percentage of intermediate (CD14^++^CD16^+^) pro-inflammatory monocytes; (2) increased activation of CD4^+^ T-lymphocytes, with increased expression of the early activation marker CD69; (3) increased amount of autoreactive CD4^+^CD28^−^ T lymphocytes, which contribute to progression and maintenance of chronic immune diseases; (4) increased differentiation of CD4^+^ T lymphocytes to Th follicular and Th22, as reflected by the increased expression of the transcription factors BCL6 and AHR; (5) increased activation of B lymphocytes and serum IgG; (6) increased levels of many pro-inflammatory cytokines [11]. These data suggest that in cirrhotic patients who are going to develop MHE some yet un-identified event triggers a cascade of immune responses which finally are transduced to the brain leading to the cognitive and motor alterations associated to MHE.

Rifaximin is a nonsystemic antibiotic approved in the United States to reduce the risk of overt HE recurrence. Several studies support this beneficial effect of rifaximin [12–15]. Moreover, several reports show that rifaximin is also able to improve cognitive function and driving ability in a large proportion of patients with MHE [16–18].

Several mechanisms of action have been proposed to account for the beneficial effects of rifaximin in HE and MHE, including effects on gut microbiota composition and/or function, bacterial translocation and on bile acids, inflammatory mediators or ammonia levels [19–22]. Bajaj [19, 20] proposes that rifaximin clinical activity may be attributed to effects on metabolic function of the gut microbiota, rather than a change in the relative bacterial abundance. DuPont [21] concludes that rifaximin may be best described as a gut microenvironment modulator with cytoprotection properties, and that further studies are needed to determine whether these putative mechanisms of action play a direct role in clinical outcomes.

Mencarelli et al. [22] showed that exposure to rifaximin caused a robust attenuation of generation of inflammatory mediators caused by LPS. Silencing of the human nuclear receptor pregnane-X receptor (PXR) completely abrogated these anti-inflammatory effects of rifaximin. They propose that the ability of rifaximin to activate the PXR contributes to the maintenance of the intestinal immune homeostasis.

Concerning the effects of rifaximin on cytokine levels in patients, there are reports showing a decrease of IL-6 or TNF-a [23, 24] while others show that treatment with rifaximin did not exert a significant effect on serum TNF-a or IL-6 levels [25, 26]. However, the effects of rifaximin treatment on many other cytokines have not been reported. Neither have been studied the effects of rifaximin on the immune parameters increased specifically in association with MHE mentioned above: expression of the early activation marker CD69; amount of autoreactive CD4^+^CD28^−^ T lymphocytes; differentiation of CD4^+^ T lymphocytes to Th follicular and Th22 and expression of the transcription factors BCL6 and AHR and serum IgG.

The aims of this work were to assess the effects of treating cirrhotic patients with MHE on: (1) MHE (performance in the PHES); (2) the percentage of intermediate (CD14^++^CD16^+^) pro-inflammatory monocytes; (3) expression of the early activation marker CD69 in T lymphocytes; (4) amount of autoreactive CD4^+^CD28^−^ T lymphocytes; (5) differentiation of CD4^+^ T lymphocytes to Th follicular and Th22, as reflected by the increased expression of the transcription factors BCL6 and AHR; (6) serum IgG levels; and (7) levels of some pro-inflammatory cytokines increased in MHE. We also aimed to assess if the changes in these parameters of the immune system are similar or different in patients who respond to rifaximin by improving MHE (responders) and those who remain in MHE (non-responders). These parameters were measured in 30 control subjects without liver disease, 30 cirrhotic patients without MHE and 22 patients with MHE. Patients with MHE were treated with rifaximin and the same parameters were measured again after 3 and 6 months of treatment.

## Methods

### Patients and controls

Fifty-two patients with liver cirrhosis were consecutively recruited from the outpatient clinics in the Hospitals Clínico and Arnau de Vilanova of Valencia, Spain. The diagnosis of cirrhosis was based on clinical, biochemical and ultrasonographic data. Exclusion criteria were: overt hepatic encephalopathy, recent (< 6 months) alcohol intake, infection, recent (< 6 weeks) antibiotic use or gastrointestinal bleeding, recent (< 6 weeks) use of drugs affecting cognitive function, presence of hepatocellular carcinoma, or neurological or psychiatric disorder. Thirty healthy volunteers were also enrolled in the study once liver disease was discarded by clinical, analytical, and serological tests. All participants were included in the study after signing a written informed consent. Study protocols were approved by the Scientific and Ethical Committees of both hospitals. The procedures followed were in accordance with the ethical guidelines of the Declaration of Helsinki. After a standard history and physical examination, blood was drawn for biochemical measurements. Blood ammonia was measured with Ammonia Test Kit II for the PocketChemBA system (Arkay, Inc., Kyoto, Japan). Psychometric tests, blood collection and ammonia determination were carried out on the same day. After performing the psychometric tests, patients were classified as without MHE or with MHE. MHE was diagnosed using the Psychometric Hepatic Encephalopathy Score (PHES). Patients were classified as having MHE when the score was ≤ − 4 points [27].

Twenty-two patients with MHE were treated with rifaximin (1.2 g/day, in three doses of 400 mg every 8 h) for 3 and 6 months. Blood collection and psychometric tests were performed just before treatment and at 3 and 6 months of treatment. Patients who improved MHE after rifaximin treatment, reaching a PHES higher than − 4, were considered as *Responders*, whereas patients remaining in MHE (PHES ≤ − 4) were considered as *Non*-*Responders*. Table 1 shows the composition of groups and clinical parameters.

**Table 1:** Etiology of liver disease and composition of the different groups

Parameters	Controls (n=30)	Patients without MHE (n=30)	Patients with MHE (n=22)	Patients with MHE (n=22)			
				“Responder” patients (n=13)		“Non-responder” patients (n=9)	
				Before rifaximin	After rifaximin treatment	Before rifaximin	After rifaximin treatment
Gender (M/F)	19/11	24/6	19/3	11/2		8/1	
Age	59 ± 1	61 ± 1	63 ± 2	60 ± 2		63 ± 2	
Alcohol		19	12	7		5	
HBV/HCV		9	5	4		1	
Others		2	5	2		3	
Ascites		2	6	2	2	1	1
Child Pugh A/B/C		26A/4B	13A/6B/3C	9A/4B	8A/5B	6A/2B/1C	5A/3B/1C
MELD		10 ± 1	11 ± 1	10 ± 1	10 ± 1	11 ± 2	12 ± 2
Haemoglobin (g/dL)	14.6 ± 0.2	13.7 ± 0.5	13.1 ± 0.4	13 ± 0.6*	13 ± 0.7*	13.7 ± 0.4	13.4 ± 0.7
Total bilirubin (mg/dL)	0.5 ± 0.03	1.3 ± 0.2**	1.2 ± 0.2*	1.1 ± 0.2*	1.2 ± 0.1*	1.3 ± 0.4*	1.5 ± 0.5*
Albumin (g/dL)	4.5 ± 0.1	3.8 ± 0.2**	3.7 ± 0.1***	3.7 ± 0.2**	3.8 ± 0.2**	3.7 ± 0.2**	3.6 ± 0.4**
ALT (U/L)	23 ± 2	36 ± 3*	33 ± 3	32 ± 4	27 ± 4	29 ± 3	34 ± 3
Sodium (mM)	138 ± 0.3	139 ± 0.7	137 ± 2	136 ± 1	139 ± 1^aa^	138 ± 3	136 ± 3^a^
Creatinin (mg/dL)	0.77 ± 0.02	0.78 ± 0.03	0.88 ± 0.05	0.84 ± 0.06	0.81 ± 0.07	0.95 ± 0.11*	0.89 ± 0.15
INR	1.05 ± 0.01	1.34 ± 0.05***	1.22 ± 0.05*	1.19 ± 0.04***	1.28 ± 0.08***	1.24 ± 0.1**	1.21 ± 0.07***
Ammonia (μM)	9 ± 1	27 ± 4*	41 ± 8***	38 ± 11**	34 ± 7*	46 ± 12***	48 ± 13***

Analytical parameters before and after rifaximin treatment in “responder” and “non-responder” patients

Values are expressed as the mean ± SEM

*MHE* minimal hepatic encephalopathy, *HBV* hepatitis B virus, *HCV* hepatitis C virus, *MELD* model end stage liver disease

Differences between controls and patients were analyzed by one-way ANOVA with post-hoc Tukey’s multiple comparison test. Differences between before and after Rifaximin treatment were analyzed using a Paired *t*-test. Values significantly different from controls are indicated by *. Values significantly different after vs. before treatment are indicated by ^a^ (*/^a^ p < 0.05; **/^aa^ p < 0.01)

### Analysis of the immunophenotype by flow cytometry

Analysis of the immunophenotype by flow cytometry was performed as in Mangas-Losada et al. [11]. 50 μL of whole blood was incubated with a mixture of monoclonal antibodies specific for the different leukocyte subpopulations (see below) and with 2 mL BD FACS Lysing Solution 1× (Becton, Dickinson and Company, Franklin Lakes, NJ, USA). Samples were incubated in the dark for 10 min at room temperature. Then, 50 µL of Flow Count (Beckman Coulter, Miami, FL, USA) was added to quantify the number of cells per microliter. Analysis was performed on a Gallios flow cytometer (Beckman Coulter, Miami, FL, USA) and the Kaluza software package was used to analyze the flow cytometry data. The cytometer settings were performed as in Balaguer et al. [28].

### Monoclonal antibodies used

Different cell populations were labeled with antibodies to CD45 (total leukocytes), CD14 and CD16 (monocytes), CD4 (T helper lymphocytes), CD28 (negative selection for autoreactive T helper lymphocytes), and CD69 (activated lymphocytes).

The antibodies used were the following: CD45-Krome Orange (clone J.33) (CD45-KO), CD4-PhycoerythrinTexas Red-X (Clone SFCI12T4D11 (T4)) (CD4− ECD), CD16-Allophycocyanin-Alexa Fluor 750 (clone 3G8) (CD16-APC-AlexaFluor750) from Beckman Coulter (Miami, FL, USA) and CD14-Pacific Blue (clone M5E2) (CD14-PB), CD28-Pacific Blue (clone CD28.2) (CD28-PB), CD69− Phycoerythrin (clone FN50) (CD69-PE).

### Determination of cytokine levels in serum

Serum or plasma samples were immediately separated and kept at − 80 °C for subsequent cytokine analysis. Concentration of IL-6, IL-18, IL-17 (high sensitivity kit), IL-21, IL-22 (Affymetrix eBioscience, Vienna, Austria), IL-15, CCL20, CXCL13 and CX3CL1 (R&D Systems, Minneapolis, MN, USA) were measure by ELISA according to the manufacturer’s instructions.

### Analysis of transcription factors by quantitative PCR

CD4^+^ T lymphocytes may differentiate into different subsets characterized by the expression of specific transcription factors. To characterize the Th and iTreg cell subsets present in patients with and without MHE treated or not with rifaximin, we analyzed the key transcription factors BCL6, AHR, TBX21, GATA3 and RORC, characteristic for Th follicular (Tfh), Th22, Th1, iTreg and Th17 cells, respectively. The most sensitive procedure to analyze them is to quantify by PCR the amount of the corresponding mRNAs in PBMCs. RNA was extracted from peripheral blood mononuclear cells (PBMCs) with an RNAspin Mini RNA Isolation Kit according to the manufacturer’s directions (GE Healthcare, Buckinghamshire, UK). The quality of RNA was checked by spectrophotometry and samples with a ratio of 2.0 for absorbance at 260 nm relative to that at 280 were selected to generate cDNA. RNA was retro-transcript into cDNA in one step with the High-Capacity RNA-to-cDNA Kit according to the manufacturer’s instructions. Taqman^®^ assays labeled with FAM dye (see below) and the Gene Expression Master Mix were used for the real-time PCR (40 cycles) (all reagents were from Applied Biosystems, Foster City, CA, USA).

Taqman^®^ gene expression assays: TBX21 (Hs00203436_m1), GATA3 (Hs00231122_m1), BCL6 (Hs00153368_m1), RORC (Hs01076122_m1), FOXP3 (Hs01085834_m1) and AHR (Hs00907314_m1). ΔΔCt method was used to determine targets expression using HPRT1 (Hs02800695_m1) as a normalizer.

### Determination of IgG level in plasma by western blot

Increased activation of B lymphocytes may be reflected in increased IgG content in plasma. As a procedure to corroborate the grade of activation B lymphocytes, we analyzed the content of IgG in plasma by Western blot, the method most usually used for relative quantification of the amount of a protein in different samples. Total protein of serum samples was quantified by a standard bicinchoninic acid assay. Samples containing 30 μg of total plasma protein were subjected to electrophoresis and immunoblotting as in [29] using anti-human IgG-Alkaline phosphatase mouse monoclonal antibody (clone GG-5, 1:80,000) (Sigma-Aldrich, St. Louis, MO, USA). The images were captured using the ScanJet 5300C (Hewlett-Packard, Amsterdam, the Netherlands) and band intensities quantified using the Alpha Imager 2200, version 3.1.2 (AlphaInnotech Corporation, San Francisco).

### Statistical analysis

Values are given as mean ± standard error (SEM). Results were analyzed by one-way analysis of variance (ANOVA) followed by post-hoc Tukey test. Association between decompensation and the response to rifaximin at 3 and 6 months of treatment were analysed by Chi square test. Results before and after rifaximin treatment were analyzed using a Paired t-test. The significance level was set at p < 0.05.

## Results

Twenty-two patients with MHE, showing a PHES of − 4 or lower just before treatment, were treated with rifaximin for 6 months. The PHES and immunophenotype analyses were repeated at 3 and 6 months of treatment.

**Fig. 1:**
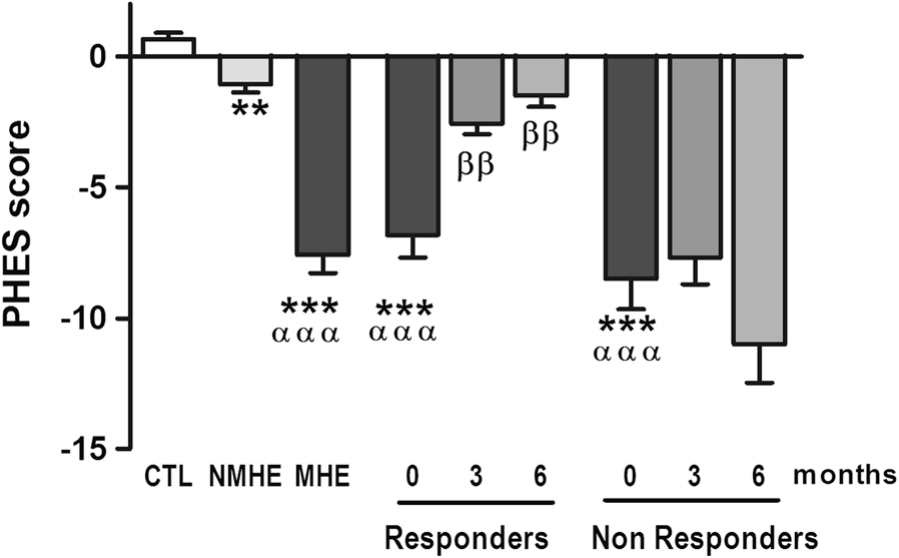
Effects of rifaximin on the PHES in responder and non-responder patients. Values are the mean ± SEM. CTL, controls; NMHE, patients without MHE; MHE, patients with MHE; 3, 6: patients with MHE after 3 and 6 months of rifaximin treatment. Values significantly different from controls are indicated by *. Values significantly different in patients with MHE compared to NMHE are indicated by α. Values significantly different after vs. before treatment are indicated by β (*/α/β p < 0.05; **/αα/ββ p < 0.01; ***/ααα/βββ p < 0.001)

Patients were considered as “responders” if after treatment with rifaximin they did not present MHE, i.e. the PHES was higher than − 4. If after rifaximin treatment the PHES remained at − 4 or lower, the patients were considered as “non-responders”. As summarized in Table 1, 13 out of the 22 patients (59%) were “responders” and improved the PHES and while 9 (41%) were “non-responders”. Analytical parameters before treatment for responder and non-responder patients did not differ significantly. No significant changes in analytical parameters (haemoglobin bilirubin, albumin, ALT, sodium, creatinine, INR or ammonia) were found after rifaximin treatment (Table 1). The PHES scores obtained by controls and by each group of patients are shown in Fig. 1.

Regarding the clinical evolution of patients during treatment with rifaximin at 3 and 6 months, some patients had decompensation (ascites, bacterial peritonitis, hepatorenal syndrome, acute-on-chronic liver failure, …) but there was no significant association between decompensation and the response to rifaximin neither at 3 nor at 6 months of treatment.

As we have recently shown that appearance of MHE is associated with specific changes in the immunophenotype [11], we assessed whether improvement of MHE by rifaximin is associated with reversal of some of these changes.

**Fig. 2:**
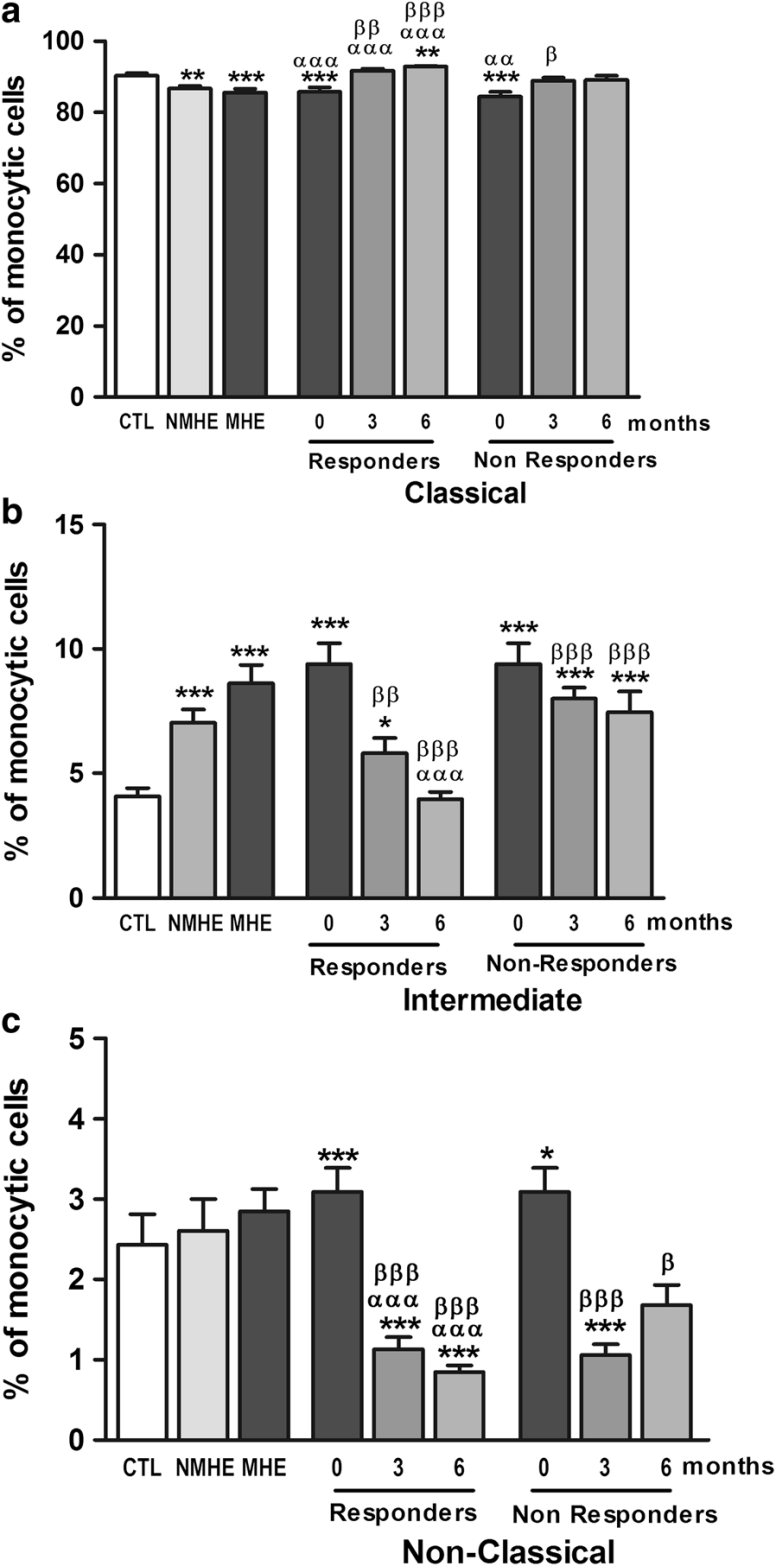
Effect of rifaximin treatment on monocytes populations in peripheral blood. Percentage of the three subsets of monocytes over total monocyte cells: **a** Classical (CD14^++^CD16^−^), **b** intermediate (CD14^++^CD16^+^) and **c** non-classical (CD14^+^CD16^++^). Values are the mean ± SEM. CTL, controls; NMHE, patients without MHE; MHE, patients with MHE; 3, 6: patients with MHE after 3 and 6 months of rifaximin treatment. Values significantly different from controls are indicated by *. Values significantly different in patients with MHE compared to NMHE are indicated by α. Values significantly different after vs. before treatment are indicated by β (*/α/β p < 0.05; **/αα/ββ p < 0.01; ***/ααα/βββ p < 0.001)

As previously reported [11], patients with MHE show an increased percentage of intermediate (CD14^++^CD16^+^) pro-inflammatory monocytes and a reduced percentage of classical, non-inflammatory CD14^++^CD16^−^ monocytes (Fig. 2a, b). Treatment with rifaximin reduced the percentage of intermediate (CD14^++^CD16^+^) pro-inflammatory monocytes, reaching normal levels at 6 months in responders (Fig. 2b). This was associated with an increase in classical CD14^++^CD16^−^ (Fig. 2a) and a decrease in non-classical CD14^++^CD16^++^ monocytes (Fig. 2c).

In non-responder patients, rifaximin also reduced the percentage of intermediate pro-inflammatory monocytes, but the reduction was milder than in responders, remaining higher than in controls (Fig. 2b).

Non-responder patients also showed a decrease in non-classical monocytes (Fig. 2c), and a slightly increase in classical monocytes after rifaximin treatment (Fig. 2a).

**Fig. 3:**
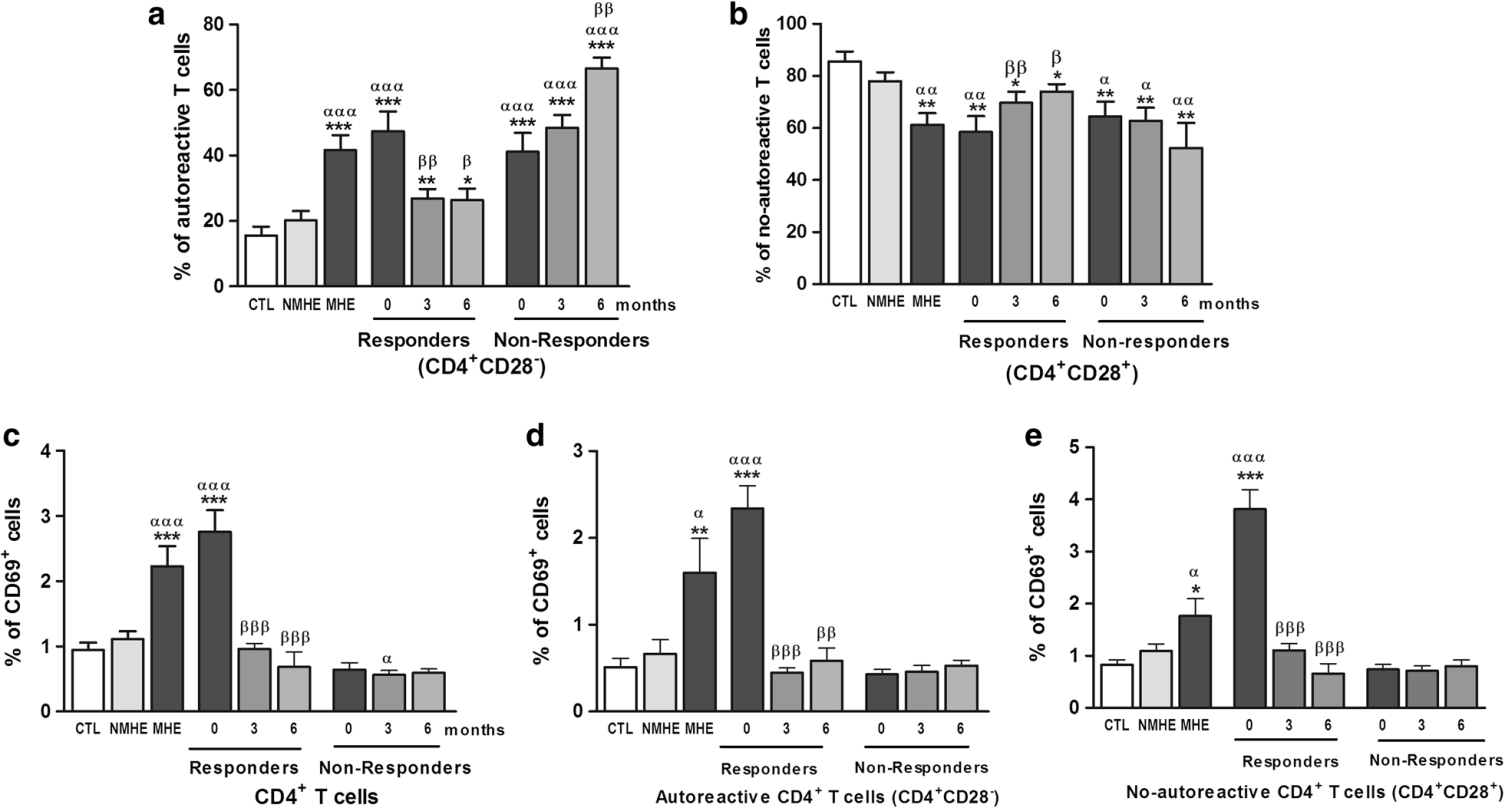
Effect of rifaximin treatment on autoreactive and no-autoreactive T lymphocytes, and on expression of the early activation marker CD69 in CD4^+^ T lymphocytes. **a** Percentage of CD4^+^ T lymphocytes that are autoreactive (CD4^+^CD28^−^). **b** Percentage of CD4^+^ T lymphocytes that are not autoreactive (CD4^+^CD28^+^). **d** Percentage of CD4^+^ T lymphocytes that express the early activation marker CD69. Percentage of autoreactive (**d**) or not autoreactive CD4^+^ T lymphocytes (**e**) that express CD69. CTL, controls; NMHE, patients without MHE; MHE, patients with MHE; 3, 6: patients with MHE after 3 and 6 months of rifaximin treatment. Values significantly different from controls are indicated by *. Values significantly different in patients with MHE compared to NMHE are indicated by α. Values significantly different after vs. before treatment are indicated by β (*/α/β p < 0.05; **/αα/ββ p < 0.01; ***/ααα/βββ p < 0.001)

Appearance of MHE is also associated with an increase in autoreactive CD4^+^CD28^−^ T lymphocytes (Fig. 3a) and a decrease in no-autoreactive CD4^+^CD28^+^ T lymphocytes (Fig. 3b). This is in agreement with the previous report [11]. Treatment with rifaximin strongly reduced autoreactive CD4^+^CD28^−^ T lymphocytes to nearly normal levels in responders but not in non-responder patients (Fig. 3a). This was associated with an increase in no-autoreactive CD4^+^CD28^+^ T lymphocytes in responders, but not in non-responder patients (Fig. 3b). In non-responders there was even an increase of autoreactive and a trend to decrease the percentage of no-autoreactive T lymphocytes at 6 months.

When all patients with MHE are considered as a whole, there is a significant increase in the percentage of CD4^+^ T lymphocytes expressing the early activation marker CD69 (Fig. 3c). The same activation pattern is observed when responder patients are considered alone. The increased expression of CD69 in CD4^+^ T lymphocytes is completely reversed by treatment with rifaximin in responder patients (Fig. 3c). In contrast, unexpectedly, non-responder patients did not show increased expression of CD69 in CD4^+^ T lymphocytes at any time, before or after rifaximin treatment (Fig. 3c). When we examined the activation of autoreactive and no-autoreactive CD4^+^ T cells, we found the same response as in CD4^+^ T cells: an increased expression of CD69 in MHE patients as a whole which was due to activation in responder patients, and a reversion by rifaximin treatment, whereas non-responder patients did not show increased expression of CD69 in these cell populations (Fig. 3d, e).

We previously reported that patients with MHE have a more potent immunological response that patients without MHE as reflected in cytokine levels in serum [11]. The increases in IL-6, IL-21, IL-17, IL-18, CCL20, CXCL13, IL-15 and CX3CL1 (fractalkine) were higher than in patients without MHE. In addition, the levels of IL-22 were increased in patients with MHE but not in patients without MHE [11].

**Fig. 4:**
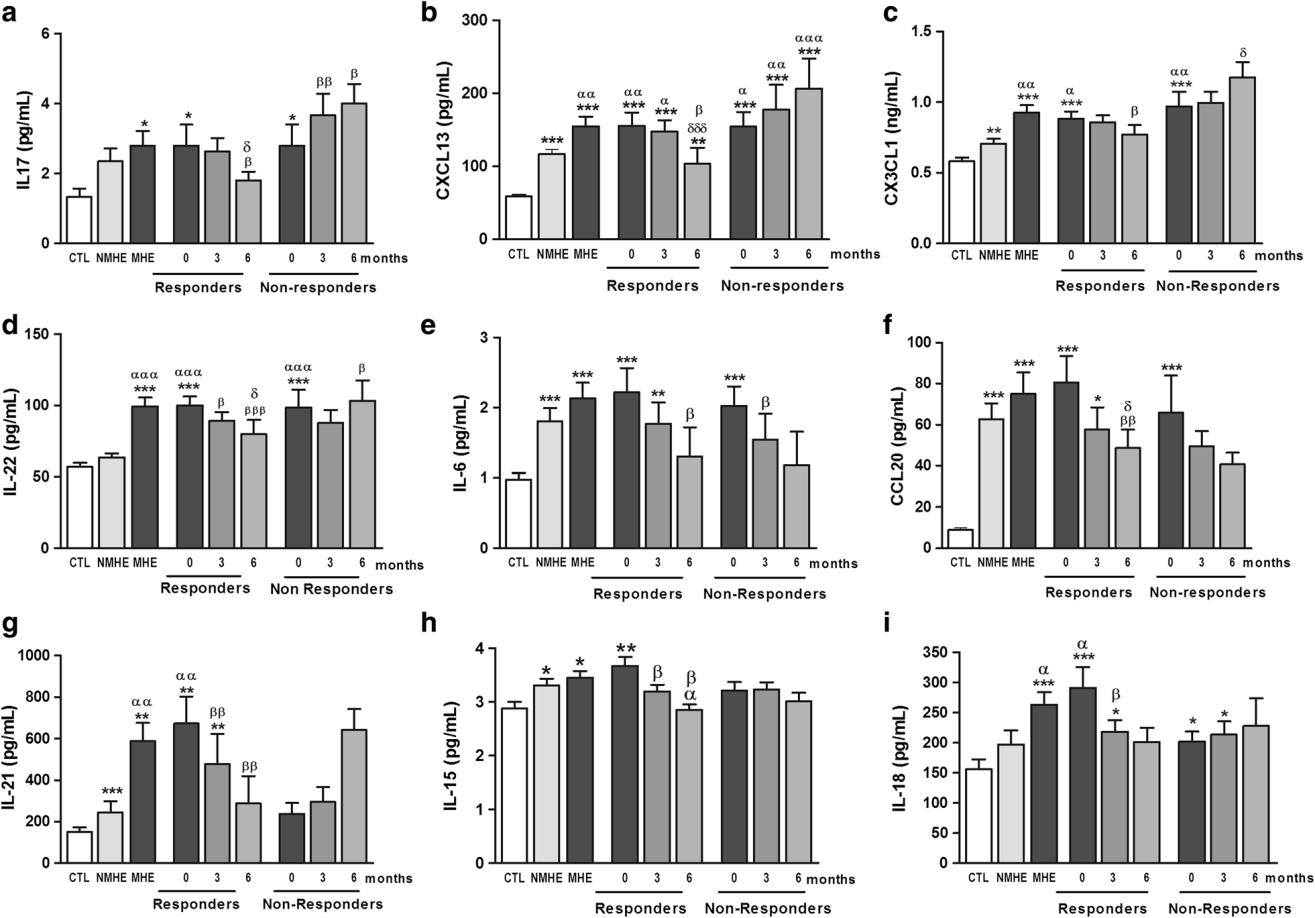
Effect of rifaximin treatment on serum levels of different cytokines. The serum levels of the indicated cytokines were measured. Values are the mean ± SEM. CTL, controls; NMHE, patients without MHE; MHE, patients with MHE; 3, 6: patients with MHE after 3 and 6 months of rifaximin treatment. Values significantly different from controls are indicated by *. Values significantly different in patients with MHE compared to NMHE are indicated by α. Values significantly different after vs. before treatment are indicated by β (*/α/β p < 0.05; **/αα/ββ p < 0.01; ***/ααα/βββ p < 0.001)

We now assessed the effects of rifaximin treatment on the serum levels of these cytokines in responders and non-responder patients (Fig. 4). We observe 3 different types of responses depending on the cytokine analyzed.

For IL-17, CXCL13, CX3CL1 (fractalkine) and IL-22 (Fig. 4a–d), treatment with rifaximin reduces the levels of these cytokines to normal values in responders but not in non-responders. Moreover, in non-responders serum levels of most of these cytokines continue increasing with time (Fig. 4a–d).

A second type of response is observed for IL-6 and CCL20, which are reduced by rifaximin both in responders and non-responders (Fig. 4e, f).

A third type of response, similar to that commented above for expression of CD69 is observed for IL-21, IL-15 and IL-18 which are increased in patients with MHE as a whole or in responder patients before treatment, but are not increased in non-responders at any time, even before treatment. As is the case for CD69, treatment with rifaximin also normalized the levels of IL-21, IL-15 and IL-18 in serum of responder patients (Fig. 4g–i).

**Fig. 5:**
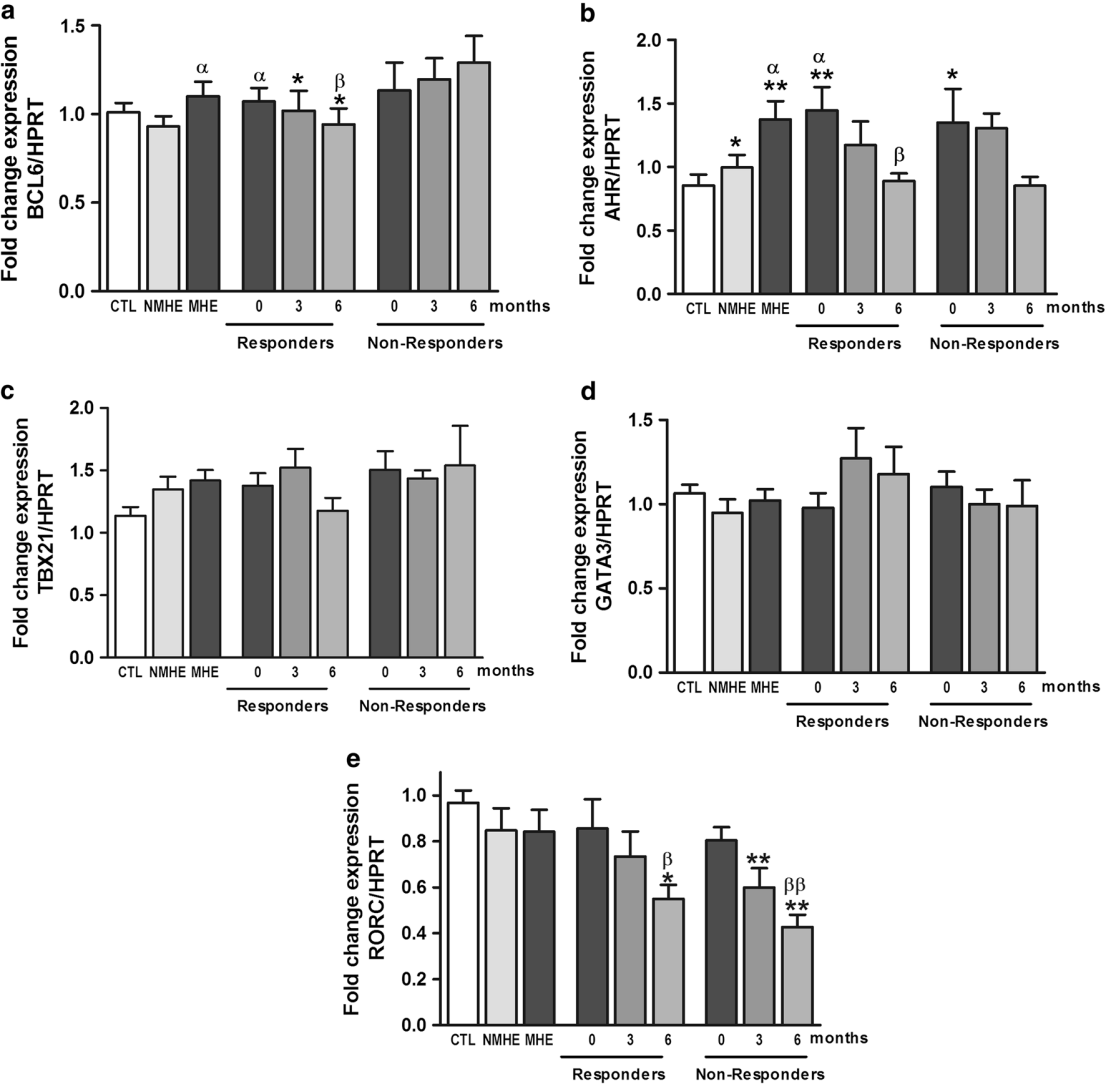
Effect of rifaximin treatment on expression of the transcription factors BCL6, AHR, TBX21, GATA3 and RORC in PBMCs. Data represent the normalized target gene amount relative to controls which are considered as 1. Values are the mean ± SEM. CTL, controls; NMHE, patients without MHE; MHE, patients with MHE; 3, 6: patients with MHE after 3 and 6 months of rifaximin treatment. Values significantly different from controls are indicated by *. Values significantly different in patients with MHE compared to NMHE are indicated by α. Values significantly different after vs. before treatment are indicated by β (*/α/β p < 0.05; **/αα/ββ p < 0.01; ***/ααα/βββ p < 0.001)

We also reported that appearance of MHE is associated with expansion of Th follicular and Th22 CD4^+^ T lymphocytes subsets, as indicated by the increased expression of the specific transcription factors BCL6 and AHR, respectively [11]. The transcription factors RORC, TBX21 and GATA3, markers of Th17, Th1 and Th2 were not altered in patients with or without MHE. We therefore also assessed the effects of rifaximin treatment on the expression of these transcription factors. Again we observed different types of responses. Treatment with rifaximin reduced the expression of BCL6 to normal levels in responders but not in non-responder patients (Fig. 5a). The expression of AHR was increased in patients with MHE and was reduced to normal values both in responders and non-responders (Fig. 5b). Expression of TBX21 (Fig. 5c) and of GATA3 (Fig. 5d) were not affected by MHE or rifaximin treatment. Finally, RORC was not affected by MHE but was reduced by treatment with rifaximin both in responders and non-responder patients (Fig. 5e).

**Fig. 6:**
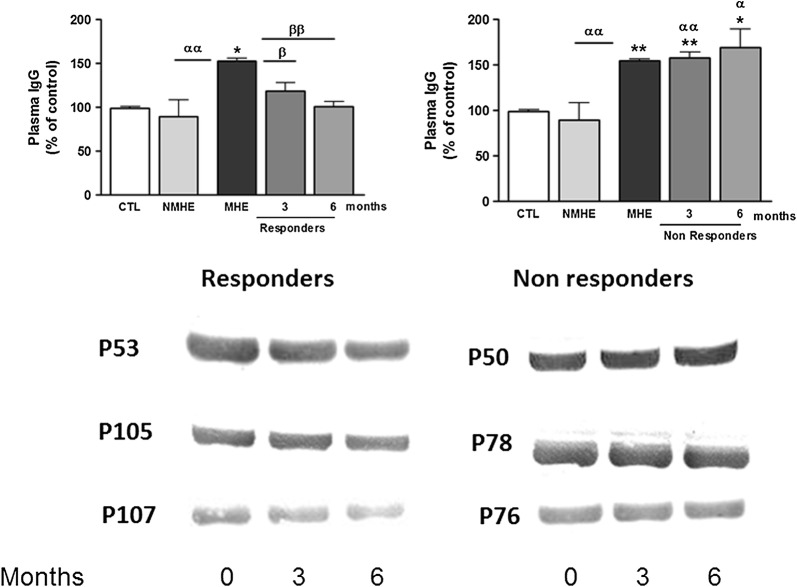
Effect of rifaximin treatment on IgG content in plasma. Content of IgG heavy (50 kDa) chain in plasma, analyzed by western blot and expressed as percentage of control subjects. Representative images of the blots are shown. CTL, controls; NMHE, patients without MHE; MHE, patients with MHE; 3, 6: patients with MHE after 3 and 6 months of rifaximin treatment. Values significantly different from controls are indicated by *. Values significantly different in patients with MHE compared to NMHE are indicated by α. Values significantly different after vs. before treatment are indicated by β (*/α/β p < 0.05; **/αα/ββ p < 0.01; ***/ααα/βββ p < 0.001)

Appearance of MHE is also associated with increased IgG levels in plasma [11]. As shown in Fig. 6, treatment with rifaximin reduced IgG levels to normal values in responder patients but not in non-responder patients.

It is noteworthy that, for many parameters, the beneficial effects of rifaximin are only partial at 3 months and are higher at 6 months of treatment.

## Discussion

In this study, rifaximin improved MHE in 13 out of 22 (59%) cirrhotic patients with MHE but not in the other 9 (41%) patients. This is in the same range reported previously in other studies on the effects of rifaximin on performance in psychometric tests and on driving ability [16–18].

The data reported support that appearance of MHE is associated with specific changes in the immunophenotype which are not present in cirrhotic patients without MHE [11].

It is noteworthy that the effects of rifaximin on immune system parameters are very different in responder patients, in which rifaximin treatment reverses MHE, and in non-responder patients. Improvement of MHE by rifaximin in responders is associated with normalization of essentially all the alterations in the immunophenotype associated to MHE. However, in non-responders, rifaximin normalizes specifically only a few parameters.

This raises a relevant question, why the same drug induces different effects in the two groups of patients? One possible explanation would be that, at the time of beginning the treatment, the alterations in the immune system are different in patients who will respond and those who will not improve MHE.

As mentioned in the introduction, the mechanisms of action of rifaximin seem to involve a modulation of the microenvironment and metabolic function of the gut microbiota, anti-inflammatory effects and maintenance of the intestinal immune homeostasis.

Rifaximin would reduce therefore pro-inflammatory factors in gut and blood. It is possible that the alterations in the immune system in responder patients are still at a stage which may be reversed by the anti-inflammatory changes induced by rifaximin while in non-responders the alterations are at a more advanced stage not reversible by rifaximin treatment. This could explain the different immunological effects of rifaximin in responders and non-responders. Moreover, if some of the alterations in the immune system are different in responders and non-responders before beginning the treatment, this could set the bases to look for predictors of the response to rifaximin in terms of improvement of MHE by analyzing a subset of immunological parameters. In fact, this report has unveiled some putative candidates to be predictors of response to rifaximin (see below).

It is also noteworthy that, in responders, rifaximin improves essentially all the immune parameters altered in association with MHE. This suggests that these changes would occur sequentially in response to some (not yet identified) common trigger of the cascade of immune alterations. To reverse the effects on all the cascade parameters, rifaximin should act at an initial or very early step of this cascade, thus reversing the immunological changes and MHE.

Rifaximin normalizes a few inflammatory factors in all patients, both in responder patients who improved MHE and in non-responder patients. The parameters that are improved in all patients are the percentage of classical and non-classical monocytes, the expression of the transcription factor AHR (a marker of Th22 lymphocytes) and the levels of IL-6 and CCL20. The normalization of AHR suggests that rifaximin reduces to normal levels the expansion of Th22 lymphocytes. IL-6 is a main promoter of the expansion of Th22 lymphocytes. It is possible that reduced expansion of Th22 lymphocytes in both responder and non-responder patients may be associated to the normalization of IL-6 levels. These data indicate that restoration of the levels of IL-6, of Th22 lymphocytes and of CCL20 is not enough to eliminate MHE when it is already present.

There is a series of immunological parameters that are improved selectively by rifaximin in patients who recover from MHE (responders) but not in non-responders, who remain in MHE. This occurs for the pro-inflammatory monocyte populations: rifaximin normalizes the levels of intermediate (CD14^++^CD16^+^) pro-inflammatory monocytes. This would reduce the pro-inflammatory environment in serum. Rifaximin also normalizes in responders, but not in non-responders, the levels of autoreactive (CD4^+^CD28^−^) and no-autoreactive (CD4^+^CD28^+^) T lymphocytes. This would also contribute to reduce the pro-inflammatory environment.

This reduction in the pro-inflammatory environment is also reflected in the selective reduction in the expansion of follicular T lymphocytes (Tfh), as reflected by the normalization of the transcription factor BCL6, and of activation of T lymphocytes, as reflected by the reduced expression of the early activation marker CD69. The reduced activation of T lymphocytes in responders would contribute to the reduced levels of pro-inflammatory cytokines such as IL-17, IL-21 and IL-22, which are produced mainly by Tfh and Th17 lymphocytes. The reduced pro-inflammatory environment in serum would also contribute to the reduced release of CXCL13 and CX3CL1 by endothelial cells and to the reduction of IgG production by B lymphocytes.

These data suggest that reversing this set of inflammatory parameters, together with the reduction of IL-6, Th22 lymphocytes and CCL20 is enough to reverse MHE and that this can be reached by rifaximin treatment in a relevant percentage of patients. This further supports a role for this inflammatory process in triggering MHE in cirrhotic patients.

The data reported also show some unexpected findings which clarification could provide tools to predict which patients with MHE would respond to rifaximin and reverse MHE and which not.

It has been found that patients with MHE as a whole show a significant increase in the expression of the early activation marker CD69 in T lymphocytes. However, when the patients are separated, after 6 months of treatment in responders and non-responders, we realized that, before rifaximin treatment, expression of CD69 is increased in responder patients who improved MHE but not in non-responder patients. Similar findings were found for the levels of IL-21, IL-15 and IL-18, which are increased in responders but not in non-responders. The reasons for this are intriguing. As CD69 is considered a marker for early activation, we may speculate that it is not increased in non-responder patients because they are not at an early phase of activation but at a more advanced phase in the immune response in which CD69 is no longer overexpressed.

CD69 is rapidly up-regulated at early stages of lymphocytes activation, for example following an acute infection [30]. Activation of lymphocytes then dramatically enhances the expression of some microRNAs such as miR130/301 which, later in the immune response, down-regulate CD69 expression [30]. CD69 expression may be also down-regulated by miR-181a [31]. Interestingly, it has been reported that miR-181b is increased in serum of cirrhotic patients [32].

Another molecule that can down-modulate surface expression of CD69 is sphingosine 1-phosphate receptor type 1 [33–35], which is involved in immune-modulation and in suppression of innate immune responses from T cells [36, 37].

We may speculate that a subset of patients with MHE (the non-responders) have progressed to a more advanced stage of the immune response in which CD69 has been down-regulated, maybe by some of the above mechanisms, and other not yet identified alterations may have occurred. At this advanced stage rifaximin would not be able to reverse most alterations in the immunophenotype nor MHE. If this hypothesis is correct, further studies to better characterize the differences in the immune system in responder and non-responder patients may allow identifying parameters useful to discriminate which patients would respond to rifaximin and which would not.

## Conclusions

In conclusion, we show that rifaximin improved MHE in 59% of patients with MHE. The effects of rifaximin on immune system parameters are very different in responder and non-responder patients. In responders rifaximin normalizes essentially all the alterations associated to MHE while in non-responders it normalizes only a few parameters. This may suggest that, at the time of beginning the treatment, the alterations in the immune system are more advanced in non-responder patients than in responders, whose alterations in the immune system would be still at a stage reversible by rifaximin. Rifaximin would recover these immune parameters by reducing the pro-inflammatory environment. These data suggest that reversing this set of inflammatory parameters is enough to reverse MHE. It is also noteworthy that for most parameters recovery at 3 months is only partial, requiring 6 months of treatment to recover. It is also shown that, before treatment, non-responder patients show differences in a few parameters compared to responder patients. They do not show increased CD69 expression in T lymphocytes nor increased IL-21, IL-15 and IL-18 levels. Further studies to better understand these differences and the underlying mechanisms may identify parameters useful to predict which patients would improve MHE in response to rifaximin and which would not.

## Data Availability

All relevant data are within the paper.

## Abbreviations

MHE: minimal hepatic encephalopathy
Th: T helper
Tfh: follicular T helper
PBMC: peripheral blood mononuclear cells
